# α7- and α9-Containing Nicotinic Acetylcholine Receptors in the Functioning of Immune System and in Pain

**DOI:** 10.3390/ijms24076524

**Published:** 2023-03-30

**Authors:** Irina Shelukhina, Andrei Siniavin, Igor Kasheverov, Lucy Ojomoko, Victor Tsetlin, Yuri Utkin

**Affiliations:** Department of Molecular Neuroimmune Signaling, Shemyakin-Ovchinnikov Institute of Bioorganic Chemistry, Russian Academy of Sciences, 117997 Moscow, Russia

**Keywords:** anti-inflammatory pathway, chronic pain, α-conotoxins, Ly6/uPAR proteins, Lynx1, nicotinic acetylcholine receptors, SLURP-1, viral infection

## Abstract

Nicotinic acetylcholine receptors (nAChRs) present as many different subtypes in the nervous and immune systems, muscles and on the cells of other organs. In the immune system, inflammation is regulated via the vagus nerve through the activation of the non-neuronal α7 nAChR subtype, affecting the production of cytokines. The analgesic properties of α7 nAChR-selective compounds are mostly based on the activation of the cholinergic anti-inflammatory pathway. The molecular mechanism of neuropathic pain relief mediated by the inhibition of α9-containing nAChRs is not fully understood yet, but the role of immune factors in this process is becoming evident. To obtain appropriate drugs, a search of selective agonists, antagonists and modulators of α7- and α9-containing nAChRs is underway. The naturally occurring three-finger snake α-neurotoxins and mammalian Ly6/uPAR proteins, as well as neurotoxic peptides α-conotoxins, are not only sophisticated tools in research on nAChRs but are also considered as potential medicines. In particular, the inhibition of the α9-containing nAChRs by α-conotoxins may be a pathway to alleviate neuropathic pain. nAChRs are involved in the inflammation processes during AIDS and other viral infections; thus they can also be means used in drug design. In this review, we discuss the role of α7- and α9-containing nAChRs in the immune processes and in pain.

## 1. Introduction

Nicotinic acetylcholine receptors (nAChRs) are pentameric ligand-gated ion channels belonging to the family of Cys-loop receptors [1]. Structural studies of nAChRs started with the muscle-type receptor from the electric organ of ray; this receptor consists of five subunits (two α1, β1, γ and δ) and is most closely related to the muscle receptors of mammals. Our review mainly deals with “non-neuronal” nAChRs, which are built from the same subunits as those in the nervous system (neuronal nAChRs) but are localized in other tissues. Nine α (α2–α10) and three β (β2–β4) subunits are currently known among these neuronal and non-neuronal subunits. Some subunits (α7, α8, α9) can be part of both homopentameric (α7, α9) and heteropentameric receptors (α7α8, α7β2, α9α10), the remaining subunits form heteropentameric channels, for example, α4β2 and α4α6β2β3.

The ligand-binding sites of all nAChRs always involve α-subunits, which have a functionally important disulfide bond formed by two vicinal cysteines in the so-called loop C, which is one of the main elements of the orthosteric binding site for agonists and competitive antagonists of all nAChRs. This disulfide is characteristic only for α-subunits, in contrast to the Cys-loop, which is present in all nAChR subunits. Orthosteric binding sites are located on the surface between the α-subunit and its adjacent subunit.

Since nAChRs have an important role in the functioning of various systems in organisms, including the immune system, and are involved in a number of pathological processes, including pain, they are considered to be promising targets for next-generation therapies. Some nAChR ligands have become medicines, e.g., the anti-smoking drugs cytisine and varenicline are already on the market; however, the treatment of immune system disorders or pain with nAChR ligands is still a challenging task. The problem is that there is a number of different nAChR subtypes, and they are present in many systems and organs; thus, their activation or inhibition may result in adverse effects. This problem can be solved by the application of highly selective ligands that affect only certain nAChR subtype or subtype populations in a particular organ. Among the various subtypes, α7 nAChR has been found to be deeply involved in immune system function, and α9α10 nAChR has been considered to be involved in pain alleviation. Two main hypotheses are considered in this review:

- nAChRs of α7 subtype are involved in anti-inflammatory pathways;
- α9α10 nAChRs participate in pain relief mechanisms.

Many of the data available in the literature are in favor of these hypotheses and are considered in this review. We try to provide current information on the compounds affecting the α7 and α9α10 nAChRs and discuss the animal toxins of α-conotoxins and α-neurotoxins, which are so far the most selective and efficient ligands (in general, inhibitors) of these receptor subtypes. While there is quite a lot of information available on the structure and activity of these compounds, and they are considered promising candidates for drug design, much more needs to be undertaken to turn them into medicines. The possible directions for this transformation are also discussed in this review.

Heteromeric and homomeric nAChRs, containing α7 and α9 subunits, have recently attracted the greatest research interest due to the revealed participation of these receptors in immune processes and pain, as well as due to their ability to be recognized by viruses. However, understanding the molecular mechanisms of the participation of these receptors in such processes is impossible without knowledge of their spatial organization.

## 2. Spatial Structure of Nicotinic Receptors and Their Complexes with Various Ligands

**Figure 1:**
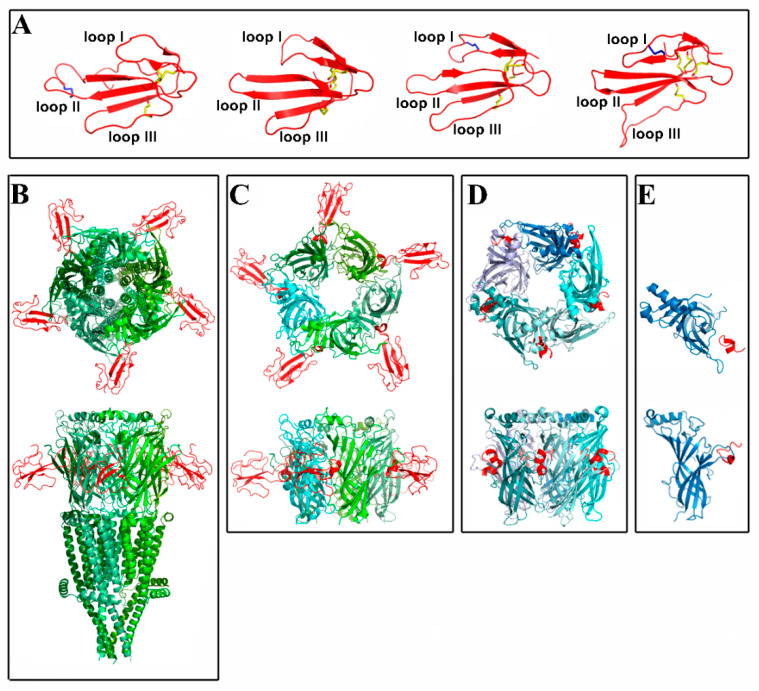
The ribbon presentation of spatial structures of three-fingered toxins as well as different complexes of the target ligand from NMR, X-ray or cryo-EM studies. (**A**) From left to right: X-ray structures of long-chain α-cobratoxin from *Naja kaouthia* (PDB ID: 2CTX) and short-chain erabutoxin-a from *Laticauda semifasciata* (PDB ID: 5EBX) as well as NMR structures of non-conventional candoxin from *Bungarus candidus* (PDB ID: 1JGK) and human water-soluble Lynx1 protein (PDB ID: 2L03). Four “core” disulfides are shown in yellow and the fifth disulfide, if any, are in blue. Three loops are indicated in all cases. (**B**) Top and side view of the cryo-EM structure of pentameric construct based on human α7 nAChR complexed with α-bungarotoxin (PDB ID: 7KOO). Identical subunits of the receptor are shown with different colors of green for clarity, and five toxins are in red. (**C**) Top and side view of the X-ray structure of pentameric chimera based on human α7 nAChR extracellular domain (ECD) and *Lymnaea stagnalis* AChBP complexed with α-bungarotoxin (PDB ID: 4HQP). Identical protomers are shown with different colors of green for clarity, and five toxins are in red. (**D**) Top and side view of the X-ray structure of *Aplysia californica* AChBP complexed with α-conotoxin PnIA analogue (PDB ID: 2BR8). Identical protein protomers are shown with different colors of blue for clarity, and five conotoxins are in red. (**E**) Top and side view of the X-ray structure of monomer of human α9 nAChR ECD (in blue) complexed with α-conotoxin RgIA (in red) (PDB ID: 6HY7).

It was a long way to the elucidation of the spatial structure of nAChRs, which started with the low-resolution X-ray structure of the nAChR from the electric ray [2], followed by low-resolution cryo-electron microscopy [3], while the next important stage was obtaining the high-resolution X-ray structure of the water-soluble acetylcholine-binding protein (AChBP) [4], which is an excellent model of the ligand-binding domains of not only nAChRs but all other Cys-loop receptors. This structure helped to establish the cryo-electron microscopy structure of the *Torpedo marmorata* nAChR, with a resolution of 4.5 Å [5]. This was then followed by the X-ray structures of the microbial Cys-loop receptors ELIC and GLIC, and only after this were several X-ray structures established for a couple of neuronal nAChRs and for their complexes with agonists and antagonists. What concerns the topics of our review, among which is brief information on such fine tools for the nAChR research, as snake venom protein neurotoxins (Figure 1A) and neurotoxic peptides α -conotoxins from *Conus* marine snails, it should be noted that there are the structures of α-bungarotoxin (a long-chain α-neurotoxin) with the *Torpedo* and α7 nAChR [6] (Figure 1B), as well as of a short-chain snake venom α-neurotoxin (which does not inhibit neuronal nAChRs) bound to the *Torpedo* receptor [7].

In the structure presented in Figure 1B, the upper part of the receptor over the membrane shows the extracellular ligand-binding domain (ECD) that includes the orthosteric ligand binding sites for agonists (acetylcholine (ACh), nicotine, synthetic compounds) and for such competitive antagonists, such as snake venom three-finger α-neurotoxins or α-conotoxins. In each receptor subunit, the intramembrane portion contains four transmembrane fragments; the major role in the organization of the channel is played by five membrane helices M2 from each of the five subunits. The lower part, the cytoplasmic domain, in all of the mentioned X-ray structures was not completely resolved, although this portion should be of functional importance for further signal transduction. For example, it is assumed that in the intracellular domain of α7 nAChR, there are binding sites for some proteins that are capable of triggering intracellular signaling cascades, which explains the metabotropic pathway of nAChR signaling in non-excitable cells, such as the cells of the immune system [8]. The first data on the structure of the cytoplasmic domain have been recently obtained through the use of ^1^H-NMR spectroscopy [9]. Several X-ray structures of complexes of various ligands with recombinant nAChR ECDs have also been obtained, for example, α7 ECD with α-bungarotoxin (Figure 1C).

## 3. Snake α-Neurotoxins and Ly6/uPAR Proteins as Research Tools and Potential Drugs

α-Bungarotoxin, a representative of three-finger snake venom α-neurotoxins, has played a crucial role in the isolation and characterization of the first individual muscle-type nAChR. α-Bungarotoxin and other so-called α-neurotoxins (Figure 1A) are still well-recognized tools in research on nAChR, helping to identify their certain subtypes in tissues and clarify their involvement in different cell mechanisms, including immunological processes and pain perception. However, they do not allow for the differentiation of certain nAChR subtypes; for example, short-type α-neurotoxins almost as efficiently as the long-chain ones inhibit or stain (as radioactive or fluorescent derivatives) the muscle-type nAChRs but are considerably less efficient in respect to α7 or α9 nAChRs compared to such long-chain α-neurotoxins, such as α-bungarotoxin or α-cobratoxin. However, a certain disadvantage of the latter two is that they have a similar high affinity for the muscle-type α7 or α9 nAChRs, thus not allowing to independently prove the presence of a particular one.

A certain role in nAChR research is also played by a group of snake toxins, which were earlier considered to be weak toxins because they are of very low toxicity, and for most of them, the targets were not known. Structurally they are very close to the long-chain α-neurotoxins, but their additional 5th disulfide is not in central loop II, but it is in N-terminal loop I (Figure 1A). One such toxin, namely WTX (weak toxin), from *Naja kaouthia* snake venom, was shown to inhibit both muscle-type and α7 nAChRs at micromolar concentrations [10] and because of its low toxicity, may be considered for possible medical applications. Recent cryo-electron microscopy determination of the WTX structure in complex with the extracellular domain of the α7 nAChR [11] may open the way for the design of medically useful variants.

In connection with the WTX and other non-conventional toxins (such as candoxin) acting on the nAChRs [12], it is appropriate to consider other groups of three-finger proteins with the same disposition of disulfides, namely some proteins of the Ly6/uPAR family that also have a 5th disulfide in loop I. Many of them are known to be present in the immune system, but the similarity of some of them to the three-finger proteins from snake venom became obvious only after its discovery in the mammalian brain of protein Lynx1 (where Ly is from the Ly6 family and nx from neurotoxin) [13]. This protein is attached to the membrane by the glycosylphosphatidylinositol (GPI) anchor and was shown to affect the functioning of several subtypes of nAChRs. At about the same time, a secreted form (having no GPI tail) named SLURP-1 (lymphocytic antigen-6/urokinase type plasminogen activator receptor-related peptide 1) was found in mammalian urine [14] and also shown to interact with nAChRs [15]. Information about these proteins can be found in several reviews [16,17,18], and here we will consider those recent publications devoted to the action of these proteins on the nAChRs, which more or less directly affected the functioning of the immune system or pain transmission.

It should be emphasized that the first effects on nAChRs were reported for overexpressed Lynx1 containing the GPI anchor, and only later wsLynx1 (water-soluble Lynx1), devoid of this tail, was prepared in *E. coli*. Its three-finger structure was confirmed by ^1^H-NMR spectroscopy, and inhibition at 10 µM at the orthosteric sites of the *T. marmorata* nAChR and of the α7 nAChR via allosteric sites were demonstrated [19]. In the case of SLURP-1, the products obtained in different laboratories had various added fusion parts, and for them was reported either agonistic activity against α7 nAChR [15] or inhibition by an allosteric mechanism with SLURP-1, which differed from the naturally occurring product by only one additional N-terminal Met residue [20]. In one case, the product identical to the native SLURP-1 was prepared through peptide synthesis and shown to inhibit several neuronal nAChR subtypes, including α9α10 nAChR [21], which at present, is considered an appropriate target against neuropathic pain. 

Endogenous SLURP-1 is believed to be an immunomodulatory protein [22]. It is known to be present in keratinocytes, and its mutations are associated with the skin disease Mal de Maleda, the reasons being mainly its interactions with α7 nAChR and some other nAChR subtypes. There is evidence that SLURP-1 facilitates the functional development of T-cells and increases ACh synthesis [23]. It was earlier shown that SLURP-1 decreased the production of TNFα by T-cells and downregulated IL-1 β and IL-6 secretion by macrophages [24]. SLURP-1 decreased the production of the inflammatory cytokines induced by TNFα [25]. It was recently detected on the mast cells, and the obtained results demonstrated that, in general, the activation of certain nAChR subtypes in the cholinergic system plays a role in the regulation of stress-sensitive inflammatory responses but may have a surprising pro-inflammatory effect on healthy skin, driving IL1β expression if SLURP-1 is involved [26]. 

It should be emphasized that, at present, there are ^1^H-NMR data for a number of those Ly6/uPAR proteins capable of interacting with nAChRs; in particular, it was shown that recombinant SLURP-1 contains two isomers and is very conformationally mobile [27].

The work on Lynx1 was performed along two lines: analyzing the effects of endogenous Lynx1 (containing the GPI anchor) or testing the effects of the added wsLynx1. Thus, experiments in mice demonstrated that the antinociceptive effects of nicotine and epibatidine were enhanced in the mice with the knockout (KO) of the Lynx1 gene. Experiments with selective antagonists revealed that here the main target should be the α4β2 nAChRs [28]. First, it was shown that wsLynx1 inhibited *T. californica* nAChR by binding at the orthosteric sites and inhibited α7 nAChRs by attaching at the allosteric sites [20], the IC_50_ values in both cases being around 50 µM. However, recently, it was found that at 2 µM, wsLynx1 increased the ACh-induced currents in rat neurons, which might be used for designing drugs against neurodegenerative diseases [29]. In the same publication, the authors provided some indications that endogenous Lynx1 has an opposite action on the activation of α7 nAChRs, which is in accord with the results produced by other laboratories. It should be noted that some of the inhibiting activities of wsLynx1 and of the other proteins of the Ly6/uPAR family expressed in *E.coli* can be reproduced by their synthetic fragments with stabilized spatial structures, which can open new pathways to drugs; in particular, the fragment of the central loop of wsLynx1 had the same capacity to inhibit the muscle-type nAChR of *T. californica* as wsLynx1 [30,31]. In conclusion, it should be mentioned that there are almost no publications where the interaction of the Ly6 proteins with the nicotinic receptors would be shown to affect the pain signal transmission, but because recent data demonstrate the activity of SLURP-1, wsLynx1 and some other water-soluble forms of the Ly6 proteins against different cancer cells [32,33,34,35] it might be expected that such proteins produce some analgesic effects as well. 

## 4. α-Conotoxins in Distinguishing the Individual nAChR Subtypes

α-Conotoxins, short neurotoxic peptides found in venoms of *Conus* mollusks targeting different nAChR subtypes, are the most accurate tools, allowing not only for distinguishing muscle-type nAChRs from neuronal ones but also the most precise identification of individual subtypes of neuronal nAChRs. There are many recent reviews covering this field (see, for example, [36,37,38]); thus, here we will briefly present information relevant to the α7 and α9α10 nAChRs, which are the focus of our review. Contrary to the complexes with the snake venom three-finger protein neurotoxins, there are not yet X-ray or cryoEM structures of α-conotoxins bound to whole-size nAChRs; the information related to the binding sites is based mainly on the X-ray structures of α-conotoxin complexes with AChBPs (see, for example, reviews [39,40], Figure 1D).

The first discovered α-conotoxin acting on neuronal nAChRs was α-conotoxin ImI. However, it showed multiple specificities, interacting not only with the α7 and α9 subtypes [41] but also with different heteromeric neuronal nAChRs [42,43]. In subsequent years, a number of new peptides interacting with α7 nAChR, namely α-conotoxins ImII [44], EpI [45], PnIB [46], AnIB [47], GID [48], OmIA [49], Vc1.2 [50], RegIIA [51], LsIA [52], CIB [53], G1.5 [54], MrIC [55], Lo1a [56], BnIA [57] and AusIA [58] were purified from venoms or derived from mRNAs isolated from the poisonous ducts of various mollusks. However, all of them either also showed multiple specificities or, similar to the last four, had a low affinity for the α7 receptor. α-Conotoxins ArIA and ArIB proved to be the most potent (although also non-selective) ligands with nanomolar affinity for α7 nAChR [59]. The situation with selectivity was partially solved by obtaining a large number of analogs of naturally occurring α-conotoxins. Thus, more potent and/or selective (towards α7 nAChR) analogs of α-conotoxins ImI [60] and PnIA [46,61,62], as well as species-selective (human/rat) peptides based on α-conotoxins RegIIA, TxIB [63] and LvIB [64] were designed and synthesized. Some of the most successful in this direction were analogs of α-conotoxin ArIB. The double mutation [V11L, V16A] increased potency (IC_50_ 0.36 nM) and another double-mutated [V11L, V16D] analog became the most specific and sufficiently active (IC_50_ 1.1 nM) ligand for homomeric α7 nAChR [59,65]. On the basis of the first of them, radioactive and fluorescent forms were prepared, which were successfully used for detecting α7 nAChR on various cellular preparations [66,67]. Currently, α-conotoxin ImI, analogs of PnIA and ArIB, are most often used to study the role of α7 nAChR in different cells and in various physiological processes, including inflammatory ones [68,69,70].

The list of conotoxins targeting α9α10 nAChR is also being updated, starting with the above-mentioned non-selective α-conotoxin ImI. In the 2000s, the α-conotoxins Vc1.1 [71,72], PeIA [73] and RgIA [74], most commonly used in studies on α9α10 receptors, were discovered. The ability to interact with this nAChR subtype was also revealed for the later-discovered α-conotoxins Vc1.2 [50], Mr1.7 [75], GIB, G1.5 [54] and Mr1.1 [76]. However, with the exception of RgIA, all other peptides showed multiple selectivities. For α-conotoxin RgIA, the X-ray structure has been established in a complex with the extracellular ligand-binding domain of the α9 subunit of human nAChR [77] (Figure 1E). It revealed the important residues of conotoxin, but it should be kept in mind that this domain is monomeric and has only one binding surface.

The particular interest in peptides targeting α9α10 nAChR was due to their revealed ability to inhibit neuropathic pain in in vivo rodent models. This was shown primarily for α-conotoxins Vc1.1 and RgIA [72,78]. However, the involvement in this analgesic effect of α9α10 nAChR was disputed [79], pointing to the inhibition of N-type calcium channels via GABA-B receptor activation [80,81]. In addition, it was revealed that the affinity of α-conotoxins Vc1.1 and RgIA to human α9α10 nAChR is significantly lower than that of rats [82,83]. All this led to the design of analogs that are highly selective toward human α9α10 nAChR. In particular, several potent and selective analogs of α-conotoxins Vc1.1 [84] and RgIA [85,86,87] were synthesized. One of the analogs of RgIA, namely RgIA4, prevented chemotherapy-induced neuropathic pain mediated through action on α9α10 nAChR; therefore, it is now considered a possible analgesic [88].

It is worth mentioning that the ability to interact with α9α10 nAChR was also shown by representatives of another group of conotoxins, namely the αO-conotoxins GeXIVA [89] and GeXXVIIA [90], αS-conotoxin GVIIIB [91] and αD-conotoxin Lt28.1 [92]. The most promising among them is αO-conotoxin GeXIVA, which exhibits high selectivity when targeting this receptor subtype with nanomolar affinity. In addition, in experiments on rodents, analgesic effects were demonstrated, mediated by the interaction with α9α10 nAChR [93,94]. This peptide contains 22 amino acid residues, being unusually long compared to typical α-conotoxins. Moreover, contrary to α-conotoxins acting on the α9α10 nAChRs via the receptor orthosteric binding sites, αO-conotoxin GeXIVA inhibits its target by attaching to some allosteric sites [89]. Another untypical feature of this conotoxin is the large number (9) of arginine residues. This was the reason for the synthesis of oligoarginine peptides of different lengths and testing their activity against nAChRs. It was found that, depending on the peptide length, oligoarginines with different efficiencies inhibited various nAChR subtypes. The highest activity (in the nanomolar range) was observed for the hexadecaarginine peptide R16 and octaarginine R8, the latter being the most efficient against the α9α10 nAChR [95]. Recently, the action of a series of oligohistidines, oligolysines and oligoarginines was tested against nAChRs, oligoarginine R9 having a higher affinity than R8 toward α9α10 nAChRs [96]. Here, it should be noted that oligoarginines were known mainly as a means for the intracellular delivery of various compounds attached to them. There are also data showing that oligoarginines can bind to the NMDA receptors and serve as potential means against neurodegenerative diseases [97], but their binding to the nAChRs was demonstrated in [95] for the first time. A comparison of the effects of R8 and α-conotoxin RgIA against neuropathic pain in a mouse model will be considered later in this review.

## 5. Functional Roles of nAChR in Immune Cells

### 5.1. Non-Neuronal Cholinergic Anti-Inflammatory Reflex

Vagus nerve signaling is an important part of the afferent loop that modulates responses to systemic endotoxemia. Efferent vagal signaling can promote the release of lymphocytes from the thymus via nAChRs [98]. In addition, the activation of the vagus nerve by endotoxin or cytokines stimulates anti-inflammatory responses. ACh is the main mediator of the vagus nerve. It has previously been found that exposure to ACh on lipopolysaccharide-activated human macrophages results in the significant inhibition of the release of pro-inflammatory cytokines, such as tumor necrosis factor (TNF-α), interleukin (IL)-1b, IL-6 and IL-18 but not of the anti-inflammatory cytokine IL-10 [99]. These results showed that the vagus nerve, previously thought to be activated only in response to peripheral inflammation, is also capable of modulating inflammatory responses. The mechanisms underlying the interaction between the nervous and immune systems were later called the cholinergic anti-inflammatory pathway (CAP) [100]. This pathway plays a significant role in controlling the inflammatory process by interacting with the nAChRs expressed on macrophages.

Vagal modulation of immune responses can occur in the densely innervated gastrointestinal tract. The parasympathetic nervous system can also control immune responses to commensal flora and food components [101]. The dietary intake of fats stimulates the production of cholecystokinin (CCK), which is a neuropeptide essential for triggering several digestive functions, including exocrine pancreatic secretion and the activation of afferent vagus nerve signals. Studies have shown that CCK released from high-fat enteral nutrition inhibited the hemorrhagic-shock-induced release of TNF-α and IL-6 [102]. This anti-inflammatory effect of CCK is mediated by the vagus nerve, as surgical or chemical vagotomy abolished the anti-inflammatory effect of both the high-fat diet and CCK administration. Accordingly, the activation of the vagus nerve prevents surgically induced inflammation of the intestinal muscles and improves postoperative ileus [103].

The identification of major macrophage-expressed receptors involved in the CAP was initially a major challenge. Macrophages are the main cells producing TNF-α [104]. Studies using genetic knockout technology and α7 nAChR-deficient mice showed that TNF-α levels in such mice were significantly higher after endotoxin administration than in wild-type mice. Similarly, electrical stimulation of the vagus nerve in α7-deficient mice did not lead to a decrease in serum TNF-α levels during endotoxemia, while in wild-type mice, a significant decrease in the level of TNF-α was observed [105]. Thus, it was concluded that the inhibition of TNF-α mediated by the vagus nerve in vivo depends on α7 nAChRs.

Further animal studies have shown that splenic nerve stimulation also inhibits TNF-α production [106]. The α7 nAChR agonist choline attenuated serum TNF-α levels in mock mice but not after splenic neurectomy [107,108]. These results suggest that α7 nAChR is a postsynaptic receptor required for splenic nerve activation and for controlling inflammatory responses. The research results show that the vagus nerve interacts functionally with the splenic nerve [108]. Electrical stimulation of the rat splenic nerve, as well as the activation of macrophage β-adrenergic receptors by norepinephrine, reduced LPS-induced TNF-α release. In this case, ACh released by the vagus nerve leads to the activation of the α7 nAChRs expressed in the ganglia of the mesenteric plexus and modulates the function of the splenic nerve through the release of norepinephrine in the spleen. The elucidation of the molecular mechanism involved in the splenic nerve in the CAP showed that that splenic nerve endings positive for β2-adrenergic receptor form synapse-like structures on T lymphocytes containing choline acetyltransferase (ChAT^+^) and synthesizing ACh – agonist of α7-receptors [109,110]. Thus, ChAT^+^ T-lymphocytes were an important intermediate step between the splenic and vagus nerve, which was necessary for the inhibition of the endotoxin-induced release of TNF-α via α7 nAChR expressing macrophages [98].

It is becoming clear that ACh is an important component of CAP, which regulates various immune processes. It has been established that various types of immune cells, such as macrophages, dendritic cells and T- and B-cells, produce ACh, which, by activating α7 nAChR, triggers an anti-inflammatory immune response [111,112]. Immune cells possess all the components of an independent cholinergic system, suggesting that ACh synthesized by immune cells plays a key role in the regulation of immune function [113]. Neuroimmune interactions mediate various functional and biochemical effects through AChRs expressed on immune cells. The importance of various components of CAP in immune cells is discussed below.

### 5.2. T-Cells

Fujii et al. [114] showed that the activation of the human T-cell lines Molt-3, CEM and HSB-2 by phytohemagglutinin (PHA) leads to the accumulation and release of ACh. The results obtained suggest that the ACh released by T-cells interacts with cholinergic receptors, thereby leading to the modulation of the immune system. In mononuclear human lymphocytes, PHA stimulation resulted in the induction of ChAT mRNA, leading to the synthesis of ACh [115]. The expression of α9 and α10 subunits has been demonstrated in T- and B-cell line but no ion currents have been reported in response to ACh [116].

Further studies identified that TCR/CD3 (T-cell receptor)-mediated T-cell activation increased ChAT expression and ACh synthesis [117]. The study of the role of the cholinergic system in Crohn’s disease showed the presence of ChAT^+^ T-cells in the spleen and Peyer’s patches of the small intestine, which are capable of synthesizing ACh and contribute to the alleviation of colitis [118]. Activated spleen cells from α7 receptor knockout mice produced higher levels of TNF-α, interferon (IFN)-γ and IL-6 [119]. The presence of functional nAChRs, including the α7 type, in human T-cells, has been confirmed. The activation of nAChR in human T-cells with nicotine led to an increase in FasL expression and the transition of cells to the G0/G1 phase [120]. Treating dendritic cells with nicotine resulted in their low ability to stimulate antigen-presenting cell-dependent T-cell responses [121]. 

A study of the role of α7 nAChRs expressed on T-cells and antigen-presenting cells (APCs) caused by GTS-21, a selective partial agonist of α7 nAChR, showed that the stimulation of α7 receptors suppresses the development of CD4^+^ T-cells, reduces the ability to present antigens, and also enhances the differentiation and proliferation of both regulatory T-cells (Tregs) and effector T-cells via the activation of JAK2/STAT signaling pathways [122]. Another study also demonstrated that nicotine reduced T-cell proliferation as well as Th1 cytokine production and facilitated the transition to Th2 but had no effect on T-cells derived from α7^−^/^−^ mice [123]. The immunosuppressive function of CD4^+^CD25^+^ Treg lymphocytes mediated by the activation of the α7 receptor has been established. The stimulation of mouse Treg lymphocytes with nicotine increased the expression of cytotoxic T-lymphocyte-associated antigen (CTLA)-4 and transcription factor p3 (Foxp3). At the same time, these effects were stopped by the selective α7 nAChR antagonist, α-bungarotoxin [124]. The treatment of mouse Treg cells with nicotine resulted in the suppression of TGF-β1 production but did not affect cell proliferation and IL-10 release, being mediated by α9 nAChR [125].

Recent studies have shown that the activation of α7 nAChR on CD4^+^ lymphocytes by GTS-21 may promote the transcription of HIV-1 proviral DNA. The activation of the α7 receptor increased the production of reactive oxygen species, decreased DUSP1 and DUSP6 and increased p38 MAPK phosphorylation [126].

### 5.3. B Cells

B-cells express ChAT and produce ACh, influencing the functions of the innate immune response [127]. Various subtypes of nAChRs are expressed in murine B lymphocytes, including α4, α5, α7, β2 and β4 nAChR subunits. The expression of α4, α7 and β2 subunits was found to be significant for the maturation of B-cells [128]. Knockout mice lacking β2 or α7 subunits had a reduced number of B-lymphocytes, and their treatment with nicotine resulted in a marked increase in the number of B-cells in the bone marrow [129]. The deficiency of α4, α7 and β2 containing nAChRs in B lymphocytes affected the rate of switching from IgM production to IgG production. The CD40-stimulated activation of B-lymphocytes derived from β2^−^/^−^ mice was more sensitive to anti-CD40, while nicotine attenuated the anti-CD40 response [130]. The proliferation of unstimulated B lymphocytes in the presence of the α7 nAChR antagonist methyllycaconitine (MLA) was enhanced, with simultaneous inhibition of LPS-induced IgM production. B-cells from α7^−^/^−^ mice produced fewer Foxp3^+^ cells, and their induction in wild-type mice, as well as IL-10 production, was inhibited by α7 nAChR ligands [131]. Taken together, these studies demonstrate that nAChRs regulate mouse B-lymphocyte proliferation and Ig production, pointing to the importance of the cholinergic regulation of the humoral immune response and immunosuppression.

### 5.4. Dendritic Cells (DC)

Dendritic cells are APCs of the immune system, causing the activation of T-cells and differentiating them into functionally different Th1- and Th2-type cells [132]. The effect of nicotine on DC resulted in the reduced production of IL-1β, IL-10 and TNF-α, especially IL-12. DC-mediated T-cell proliferation and IFN-γ release were reduced. Nicotine had a direct effect on the ability of DC to polarize Th1 cells, indicating reduced immunostimulatory functions of DC [121,133]. CD205^+^ DC secrete SLURP-1. Recombinant SLURP-1 attenuated the proliferation of peripheral blood mononuclear cells and increased the amount of ACh in the Molt-3 cells. The selective α7 nAChR antagonist MLA abolished these effects, suggesting an α7-receptor-dependent modulation of immune responses by SLURP-1 [134]. The α7 nAChR agonist GTS-21 improved the clinical course of arthritis in a mouse model of collagen-induced arthritis (CIA) by reducing the secretion of pro-inflammatory cytokines and downregulating CD80 and MHC II expression on the DCs of mice [135]. GTS-21 suppressed the APC-dependent differentiation of CD4^+^ T-cells in Tregs. Ovalbumin-induced release of IFN-γ, IL-4 and IL-17 from the spleen cells of TCR transgenic mice was reduced by GTS-21. However, antagonists of α7 nAChRs (α-bungarotoxin and MLA) did not abolish the effects of GTS-21, which may indicate a pharmacological difference between non-neuronal α7 nAChRs expressed on DCs and those in neurons [136]. DC polarization under the influence of ACh led to the stimulation of OX40L expression, the induction of the Th2 profile and increased production of IL-4, IL-5 and IL-13 by CD4^+^ T-cells [137]. Nicotine exposure to mouse and human DCs resulted in the increased expression of CD86 relative to CD80 and in the production of less IL-12, modulating the Th1/Th2 balance toward Th2 [138]. In a mouse model of sepsis-induced acute lung injury (ALI), GTS-21 reduced DC maturation and the production of pro-inflammatory cytokines, thereby reducing inflammatory responses in ALI [139].

### 5.5. Monocytes

Monocytes play an important role in the primary innate immune response. The human monocytic cell line U937 expresses various subtypes of nAChRs. In LPS-stimulated U937 cells, the nAChR agonist epibatidine inhibited the production of pro-inflammatory cytokines (TNF-α, IL-1β, IL-6 and IL-18) [140]. The treatment of monocyte THP-1 upon LPS activation with nicotine or the specific α7 nAChR agonist GSK1345038 resulted in the inhibition of TNF production [141]. The exposure of the primary human monocytes to nicotine resulted in the inhibition of the IL-18-enhanced expression of ICAM-1, B7.2 and CD40 and in the production of IL-12, IFN-γ and TNF-α by lymphocytes. The nonselective nAChR antagonist mecamylamine and selective for α7 nAChR α-bungarotoxin abolished the effects of nicotine, suggesting its dependence on the α7 receptor [142]. Nicotine inhibited IL-18 and IL-12 production in LPS-stimulated monocytes [143]. The activation of α7 nAChR in mouse Ly6C^hi^ spleen monocytes by GTS-21 suppressed TNF-α production. Exposure to various TLR ligands in the GTS-21-primed J774 monocytes resulted in the mRNA inhibition of TNF-α and IL-1β expression, increased histone deacetylation and inhibited NF-kB p65. These results indicate that α7 nAChR activation confers “anti-pro-inflammatory” memory to monocytes [144]. In mouse bone marrow cells, the α7 and α9 nAChR subtypes are the most common. Nicotine reduces the total number of monocytes and inhibits the IFN-γ-induced increase in pro-inflammatory monocytes. Nicotine exposure resulted in the inhibition of the production of pro-inflammatory cytokines TNF-α, IL-1β and IL-12 by monocytes in the bone marrow while simultaneously stimulating the secretion of IL-10 [145]. Nicotine, acting on the α7 and α9 receptor subtypes, significantly inhibits the infiltration of pro-inflammatory monocytes and neutrophils into the CNS in a model of experimental autoimmune encephalomyelitis (EAE). mRNA levels for the chemokines CCL2 and CXCL2 are downregulated in the brains of nicotine-treated EAE mice prior to the massive infiltration of these cells, suggesting the importance of nAChRs in the treatment of neuroinflammatory diseases [146]. The administration of GTS-21 agonist provides effective elimination of pathogenic microorganisms, reduced inflammatory response and organ damage in the model of polymicrobial septic peritonitis. Stimulation by GTS-21 leads to the enhanced recruitment of monocytes into the abdominal cavity and to a simultaneous increase in phagocytic activity and the iNOS expression of these recruited monocytes [147].

A novel cholinergic mechanism has been discovered that inhibits the ATP-dependent release of IL-1β by human monocytes via nAChRs. It has been shown that phosphocholine (PC) is an agonist for monocytic nAChRs containing α9 and α10 subunits. PC and choline (Cho) inhibited the ATP-induced release of IL-1β from LPS-primed human monocytic U937 cells with the participation of α9 nAChR, which was confirmed by using conotoxin RgIA4, a selective antagonist of this receptor subtype [148]. At the same time, a metabotropic mechanism of action of PC on monocytic nAChR was assumed. ACh and nicotine (Nic) also completely inhibited the release of IL-1β from U937 cells primed with LPS and stimulated with BzATP. α-Conotoxin ArIB[V11L, V16D], a selective α7 nAChR antagonist, abolished the inhibitory effects of ACh and Nic [149]. Subsequently, a number of compounds were identified which, by interacting with α7 and α9 receptors, lead to the inhibition of IL-1β release: a synthetic surfactant, palmitoylphosphatidylglycerol and dipalmitoylphosphatidylcholine (DPPC), lysophosphatidylcholine (LPC) and glycerophosphocholine (G-PC), PC-modified lipooligosaccharides (PC-LOS) from *Haemophilus influenzae* and C-reactive protein [69,150,151,152,153].

### 5.6. Macrophages

The mRNA expression of the α9 and α10 nAChR subunits was found in murine alveolar macrophages. However, [Ca^2+^]i in response to nicotine and ACh was not detected [154]. The stimulation of human macrophages (MDMs), as well as the murine macrophage RAW 264.7 cell line, with cholinergic agonists, resulted in a significant decrease in TNF production. The activation of α7 nAChR induces CREB-dependent transcriptional changes in macrophages, resulting in reduced TNF production in response to endotoxins [155]. ACh attenuated the release of various pro-inflammatory cytokines in LPS-activated MDMs, such as TNF, IL-1β, IL-6 and IL-18, but not of anti-inflammatory IL-10 [99]. The functional expression of α7 nAChRs on MDMs and macrophages from the monocytic THP-1 cells has been confirmed using a selective agonist PNU 282981. The activation of α7 receptors on macrophages led to an increase in the expression of membrane proteins HLA-DR, CD11b and CD54 but to a decrease in the expression of CD14 and in the production of IL-10 [156]. The anti-inflammatory effects of nicotine in peritoneal macrophages have been associated with the activation of the Jak2/STAT3 signaling pathway [103]. In LPS-activated macrophages RAW 264.7, agonists such as ACh and PNU 282987 inhibited MMP-9 (matrix metalloproteinase) production and cell migration; ACh activated the expression of JAK2 and STAT3. At the same time, the α7-receptor antagonist MLA abolished all the effects caused by cholinergic agents in cells [157]. The activation of the α7-receptor in LPS-stimulated RAW 264.7 macrophages resulted in the inhibition of mRNA expression and the production of TNF-α, IL-6, and IL-1β. α7 nAChR antagonists, such as MLA, α-cobratoxin and α-conotoxin PnIA[A10L], showed the opposite effect and increased the production of pro-inflammatory cytokines [70].

Using a mouse model of intracerebral hemorrhage, it was found that PNU 282987 leads to a decrease in inflammatory factors and contributes to the polarization of macrophages into an anti-inflammatory phenotype. The activation of the α7-receptor promoted autophagy by increasing LC3 protein (Beclin) and the recovery of brain and heart function [158]. 

Specific α7-receptor agonists reduced the levels of the pro-inflammatory cytokines MIP-2 and TNF-α and the activation of nuclear factor NF-κB in a rodent model of acute lung injury, which was mediated by the activation of α7-receptors expressed by alveolar macrophages [159]. The administration of GTS-21 to mice with LPS-induced inflammation inhibited the production of TNF-α but not IL-6, by alveolar macrophages [160]. 

Chronic inflammation is also observed in viral infections, such as that of HIV. The CCR5 and CXCR4 glycoproteins of HIV tropic variants, such as gp120_JRFL_ and gp120_IIIB_, lead to the activation of α7 receptors on MDMs. A paradoxical pro-inflammatory phenotype was observed in macrophages, resulting in a failure to inhibit the release of pro-inflammatory cytokines, thus indicating the disruption of CAP in MDMs [161,162].

### 5.7. Mast Cells

The rat mast/basophil cell line RBL-2H3 expresses α7, α9 and α10 nAChRs. Nicotine exposure resulted in the inhibition of C4 leukotriene, TNF-α and IL-1β production after cell activation with FcεRI but had no effect on histamine release. These effects were associated with the inhibition of cytosolic phospholipase A_2_ activity and the PI3K/ERK/NF-κB pathway and were blocked by α7/α9-nAChR antagonists [163]. The treatment of rat mast cells with low concentrations of ACh caused the release of histamine [164]. The binding of fluorescently labeled α-bungarotoxin to human mast cells HMC-1 indicated the presence of nAChR expression [165]. Human mast cells contain high levels of acetylcholinesterase. Cholinergic agonists cause mast cell degranulation but do not affect leukotriene B or TNF-α secretion [166]. The treatment of mouse bone marrow mast cells with α7-receptor agonists significantly inhibited antigen-induced degranulation, indicating the downregulation of mast cell activation via α7 nAChR [167].

### 5.8. Neutrophils and Granulocytes

Mouse polymorphonuclear neutrophils express nAChRs. Nicotine and ACh modified the respiratory burst and affected neutrophil adhesion, indicating a regulatory role of α7, α3β2, or α6 receptor types in neutrophil function [168]. Nicotine, choline and other cholinergic agents induced Ca^2+^ transients in polymorphonuclear neutrophilic granulocytes, increasing cell adhesion and decreasing the production of reactive oxygen species, which was mediated by α9 nAChR [169]. The treatment of neutrophils with nicotine led to the release of neutrophil extracellular traps and the activation of enzymes Akt and PAD4 [170]. In a model of sepsis-induced acute lung injury, the administration of α7 nAChR agonists resulted in a decrease in the transalveolar migration of neutrophils [171].

### 5.9. Natural Killer (NK) Cells

Natural killer (NK) cells are effector lymphocytes that control several types of tumors and microbial infections [172]. Human NK cells express α7 nAChR. In cytokine-stimulated NK cells, the activation of α7 receptors by PNU 282987 suppressed NKG2D expression but did not affect NKp46 (CD335; cytotoxicity-activating receptor) and DNAM-1 (CD226; accessory molecule). NK cells exposed to a specific α7 nAChR agonist exhibited reduced cytotoxic activity and IFN-γ production and showed reduced p65 NF-κB nuclear mobilization. The co-cultivation of PNU 282987-treated NK cells with DC resulted in significantly lower MHC-II and CD83 expression in DCs and a lower percentage of CD86^high^ DCs [173]. Mouse NK cells express ChAT and synthesize higher amounts of ACh during inflammation. ChAT^+^ NK cells and CCR2^+^Ly6C^hi^ monocytes form immune synapses, which helps to reduce infiltration and the production of pro-inflammatory cytokines [174]. Another study reports that nicotine impairs the ability of NK cells to kill cancer cells and release cytokines. The treatment of mouse NK cells with nicotine resulted in the decreased expression of NKG2D (CD314; type II integral membrane protein), Ly49I (homodimer type II transmembrane protein) and diminished cell proliferation. NK cells exposed to nicotine were less effective at killing B16 melanoma tumor cells [175]. Rat spleen NK cells treated with ACh also showed reduced lysis of Yac-1 lymphoma cells [176].

To conclude this section, it can be said that ACh is produced by various types of immune cells and is involved in many biological processes. Agonists of nAChRs, such as ACh and nicotine, can act on different types of immune cells through autocrine or paracrine stimulation. The data related to the biological effects of the actions on the α7, α9 and α9α10 nAChRs in different immune cells are summarized in Table 1. The cholinergic anti-inflammatory pathway is an important mechanism activation of which, by nAChRs, leads to the inhibition of cytokine production. Vagus nerve stimulation of ACh release, as well as the administration of cholinergic agonists, reduce cytokine production in various models of inflammation. The activation or inhibition of nAChRs opens up new therapeutic possibilities for the effective treatment of various inflammatory, autoimmune and infectious diseases.

## 6. α7- and α9-Containing nAChRs in Chronic Pain

### 6.1. α7 nAChR in Pain

The analgesic properties of drugs selective to α7 nAChR have attracted significant attention after the discovery of the leading role of this receptor subtype in the vagal-mediated CAP (see part 5.1 of this review). The expression of α7 nAChR along pain transmission pathways has been known for a long time (reviewed in [177]). Moreover, the protein level of α7 nAChR is significantly downregulated in the sciatic nerve, dorsal root ganglion (DRG) and spinal cord in animal pain models of different etiology, providing a cellular and molecular basis for the known alleviation of chronic pain, including neuropathic pain, inflammatory pain and cancer-induced bone pain owing to activation or positive modulation of α7 nAChR (reviewed in [178]). Consistent with this was the observed decrease in the expression of α7 and β2 nAChR in the spinal cord and midbrain periaqueductal gray of hyperalgesic rats, which was reversed by analgesic electroacupuncture stimulation [179]. Potentially, not only homopentameric α7 but also heteromeric α7β2 nAChRs might be involved in pain regulation. There are some excellent recent reviews about the role of α7 nAChR and its specific ligands in pain modulation [177,178,180]. In this article, we are going to briefly review the newest tendencies and studies published in this field in the last two years.

Potent antinociceptive effects have been shown for α7 nAChR-targeting full, partial and silent agonists and positive allosteric modulators (reviewed in [177,178,180]). However, there is a tendency toward the reconsideration of the subtype-specificity of compounds acting on nAChRs. For example, among α7-selective partial and silent agonists, including those possessing anti-inflammatory and antinociceptive activity [181], some new α9-specific potent agonists and antagonists have been discovered [182]. The results of work with phosphocholine and pCF3-diEPP, silent/partial agonists of both α7 and α9-containing nAChRs, suggest that the responses to agonists by both receptor subtypes may be the basis for the observed anti-inflammatory effects in monocytic and macrophage-like cells [183].

CHRFAM7A is a relatively recent and exclusively human gene arising from the partial duplication of exons 5 to 10 of the α7 nAChR-subunit-encoding gene, CHRNA7 [184]. CHRFAM7A translates the dupα7 protein in a multitude of cell lines and heterologous systems while maintaining processing and trafficking that are very similar to those of the full-length form. Two isoforms of the CHRFAM7A gene transcripts code dupα7 proteins, which are short of a part or of the entire binding site but contain all of the α7 transmembrane domain sequences [185]. The co-expression of this gene with full-length α7 has dominant negative effects on ion channel function [186,187,188] and on the α7-nAChR-mediated control of exocytotic neurotransmitter release [189]. Through the regulation of the cholinergic anti-inflammatory pathway (reviewed in [177,190]), the CHRFAM7A gene expression level is linked to the severity of inflammatory-related pathologies, such as sepsis, osteoarthritis [191,192], cerebral ischemia/reperfusion injury, hypertrophic scars, COVID-19, inflammatory bowel disease [193], renal fibrosis [194] and pain, including neuropathic pain after spinal cord injury [195] and inflammatory pain due to osteoarthritis modeling [192].

### 6.2. α9-Containing nAChR in Pain

The first indications of the analgesic effects mediated by α9α10 nAChR arose when this receptor subtype was identified as a molecular target for analgesic α-conotoxins Vc1.1 and RgIA, which alleviated neuropathic pain in rodent models (reviewed in [196,197,198]). In particular, α-conotoxin RgIA (daily i.m. injection of 2 or 10 nmol) and its analog RgIA4 (daily s.c. injection of 0.128–80 μg/kg dose), which is highly specific to both human and rodent α9α10 nAChRs, were analgesic in oxaliplatin-induced peripheral neuropathy in rats and mice [85,86,199]. The latter also accelerated recovery from paclitaxel-induced neuropathic pain [88]. Oxaliplatin and paclitaxel are first-line platinum- and taxane-based anti-cancer drugs, efficient against colorectal, breast, ovarian and non-small cell lung cancers [200,201]. The most common side effects of such chemotherapeutics are neurological reactions, namely chemotherapy-induced peripheral neuropathy (CIPN). Characteristic manifestations of CIPN are impaired cold sensitivity (cold allodynia) as well as headache, numbness, chronic pain and asthenia [202,203,204]. Currently, there is no entirely effective protocol to treat CIPN [205]. Our recent findings have shown that another α9α10 nAChR antagonist, oligoarginine R8, a member of a new class of nAChR inhibitors [95], was as effective as RgIA in alleviating oxaliplatin-induced neuropathic pain in mice at a five-times lower dosage (0.5 nmol (20 mg/kg) of R8 versus 2 nmol (100 mg/kg) of RgIA i.m. daily administration) [206]. Another α9α10-specific αO-conotoxin, GeXIVA, also alleviates chemotherapy-induced peripheral neuropathic pain induced by oxaliplatin [93] and paclitaxel [207] at a dosage of 0.45 mg/kg i.m. GeXIVA possesses four rather than six Cys residues, which is a unique structural property among members of the O1 conotoxin gene superfamily [89].

The blockade of α9α10 nAChR also provided positive effects in a rat chronic constriction injury (CCI) pain model, another model of neuropathy. Several specific α9α10 nAChR α-conotoxins Vc1.1, RgIA, Mr1.1 [S4Dap], αO-conotoxin GeXIVA [1,2] (≤2 nmol, i.m.) and small-molecule ZZ-204G (≥3.6 μg/kg) displayed analgesic activity in a CCI pain model [72,76,78,89,94,208,209]. Thus, α9α10 nAChR antagonists are considered to have great potential in relieving the neuropathic pain induced by diabetic peripheral neuropathy, the most common complication associated with long-term diabetes mellitus [210].

Repeated treatments with conotoxins RgIA, RgIA4, GeXIVA [1,2], oligoarginine R8 and small-molecule ZZ-204G inhibitors of α9α10 nAChR produced a cumulative analgesic effect without tolerance and, in some studies, promoted recovery from neuropathic pain [85,86,88,93,94,199,206,208,209]. A number of studies indicated that therapeutic doses of these substances, which were shown to be analgesic, did not impair rodent motor functions [89,93,206,209].

#### 6.2.1. Molecular Mechanisms of Analgesia Mediated by α9-Containing nAChRs

The molecular mechanism of analgesia mediated by the inhibition of α9α10 nAChR is not fully understood yet. The analgesic effects of α9α10 nAChR inhibitors, reported for neuropathies of different origins (chronic constriction injury pain model and chemotherapy-induced neuropathy), may be partially realized by the direct antagonism of α9α10 receptors expressed in sensory DRG neurons. These neurons are pseudounipolar, with their axons outstretched to the spinal cord and periphery. DRG neurons express a number of nAChRs, including both the α9 and α10 nAChR subunits [211]. Some nAChRs have been shown to be axonally transported in DRG neurons and accumulated at the site of sciatic nerve ligation [212]. Such trauma provokes the development of neuropathic pain in CCI and PNL (partial nerve ligation) pain models. The possible involvement of peripheral neuronal α9α10 nAChRs in the analgesic effects of α-conotoxins Vc1.1, RgIA and αO-Conotoxin GeXIVA [1,2] is consistent with the observed acute analgesic effect of their intramuscular injection, developing within 1–4 h in CCI, PNL and chemotherapy-induced neuropathies in rat [72,78,93,94,207].

On the other hand, α-conotoxin treatment provokes multiple long-lasting processes. The analgesic effects of α-conotoxins Vc1.1 and RgIA administration continued for 7–12 days post-treatment in different neuropathy pain models [78,213]. The long-lasting analgesic effect of GeXIVA [1,2] on mechanical allodynia in CCI and oxaliplatin-treated rats continued for 10–14 days after the repeated intramuscular administration of the toxin was ceased [93,94]. The daily administration of RgIA4 (s.c., 40 μg/kg) reversed oxaliplatin-induced cold allodynia in mice, but only after 3 weeks of treatment [86]; in a rat paclitaxel-induced neuropathic pain model, its therapeutic effects reached significance 12 days after the last administration of RgIA4 (daily s.c. injection of 80 µg/kg for a month), which is suggestive of a rescue mechanism [88]. In addition, chronic α-conotoxins Vc1.1, RgIA and oligoarginine R8 administration led to neuroprotection, preventing CCI-induced degenerative changes both in the sciatic nerve structure [78,208] and oxaliplatin-induced DNA damage in dorsal root ganglion neurons in rat neuropathic pain models [199,206].

The observed long-lasting effects, including neuroprotection, might lead to the properties of α-conotoxins modulating the number and functioning of glial cells [199,208]. In CCI and oxaliplatin-treated rats, RgIA treatment prevented a numerical increase in microglia and astrocyte cell density present in the spinal cord, but it was able, per se, to elicit a numerical increase and morphological activation in microglia and astrocytes in specific brain areas, suggesting that RgIA may modulate glial cells in order to promote neurorestoration and reduce pain [199,208].

In addition, the role in pain relief through the actions on the α9α10 nAChRs present in the immune cells is becoming more evident. The blockade of α9α10 nAChR with α-conotoxins or small-molecule inhibitor ZZ-204G can alleviate both chronic neuropathic and inflammatory pain [209,214]. Potent α9-selective agonists, as well as numerous antagonists, have been described recently [182]. Several of these compounds have previously been shown to be effective in animal models of inflammatory pain, an activity that was assumed to be due to α7 silent or partial agonism but may, in fact, be due to α9 nAChR activity [181]. Moreover, the chronic constriction of the sciatic nerve produces an inflammatory response at the site of the injury [215]. This inflammatory response is thought to contribute to the development of neuropathic pain following peripheral nerve injury [215]. In rat CCI pain models, RgIA or Vc1.1 (0.2–10 nmol i.m.), in addition to their analgesic effects, significantly reduced edema and inflammatory infiltrate, including a decrease in macrophages and T-cells [72,208]. Recently, it has been shown that the RgIA4 prevention of acute oxaliplatin-induced cold allodynia requires both α9-containing nAChRs and CD3^+^ T-cells [216]. In this study, the subcutaneous administration of RgIA4 (40 µg/kg daily for 4 days) demonstrated an analgesic effect in wild-type mice, but not in mice lacking the α9 nAChR-encoding gene, *chrna9*, or depleted CD3^+^ T-cells. Thus, long-lasting α-conotoxin-induced analgesia may include specific activities of immune and glial cells and such α9α10 nAChR-mediated mechanisms remain an area of active investigation. 

Several studies have proposed that α-conotoxins Vc1.1, RgIA and αO-conotoxin GeXIVA can exert analgesic effects through the modulation of the N-type VGCC Ca_V_2.2 via the stimulation of G protein-coupled γ-aminobutyric acid type B (GABA_B_) receptors instead of the inhibition of α9-containing nAChR [217,218,219,220,221,222,223,224]. This is consistent with the observed inhibition of N-type VGCC CaV2.2 by α-conotoxins Vc1.1 and RgIA in DRG neurons of α9 KO mice [222]. Although such molecular mechanism of action is well-characterized for ω-conotoxin MVIIA (ziconotide), a recommended drug for first-choice intrathecal monotherapy against chronic pain [225], several studies have not fully reproduced such inhibitory effects on the high-voltage-activated calcium channels for the α- and αO-conotoxins [89,226,227,228]. Moreover, α-conotoxin RgIA4, a derivative of RgIA, lacks GABA_B_ receptor activity but maintains the capacity of the parent peptide to prevent the development of neuropathic pain [85,86,88]. The studies carried out with the α9 subunit KO mice also prove the necessity of α9-containing nAChRs for chronic pain relief [85,216,227,229].

#### 6.2.2. α-Conotoxin-Based Drug Development Strategies

Although α-conotoxins specific to α9α10 nAChR hold great pharmacological potential, they are not ideal therapeutic drug leads due to a number of shortcomings, such as a short half-life in vivo, unstable disulfide bonds, limited modes of administration and poor potency at human nAChRs for some of them [230,231,232]. In recent reviews [197,198], the established strategies for improving the activity, selectivity and stability of α-conotoxins have been considered in detail. They include scanning mutagenesis, unnatural amino acid substitutions, disulfide bond modification, backbone and side-chain cyclization and polymerization. Moreover, drug development for clinical use requires working out a stable medicinal formulation. In the case of conotoxins, lyophilization or encapsulation in microspheres could diminish their intrinsic instability and provide a sustained release of the drug to meet the clinical treatment needs of chronic pain [207,233]. In addition, new small-molecules [182,234] and peptide [235] inhibitors of α9-containing nAChRs are emerging, differing in chemical structure from known analogs. For example, a new A-superfamily conotoxin Bt14.12, similar to α-conotoxins and other A-superfamily conotoxins, contains a four Cys (C-C-C-C) framework, but with a unique disulfide bond connection “C1-C4, C2-C3”. Interestingly, the addition of three Arg residues at the N-terminus of Bt14.12 enhances its inhibitory activity four-fold compared to wild-type Bt14.12, opening new perspectives of chimeric constructions between pain-relieving α9-specific conotoxins and oligoarginines [95,206]. Moreover, the discovery of several potent α9-specific agonists [182] suggested a new future direction for analgesic drug development.

## 7. α7- and α9-Containing nAChRs as Targets in Viral Infection

Nicotinic acetylcholine receptors are involved in inflammatory processes and nociception but are also direct targets recognized by a number of viruses. The first and canonical example here was a study on the interaction of the trimeric Rabies virus glycoprotein (RVG) with nAChRs, which began in the 1980s. At first, the homology of the central loop of snake α-neurotoxins with a certain fragment of the viral protein was revealed, and then the ability of this receptor-recognizing fragment 173–204 (as well as of the full-size RVG) to interact with the orthosteric binding site of the muscle and muscle-type nAChRs was demonstrated; polyclonal antibodies to this fragment were also obtained, which bound to cholinergic ligands. Since that time, attempts have been undertaken to use the RVG fragments of different lengths both to inhibit the binding of the virus and to facilitate the delivery of various compounds to the central nervous system. It is well known that the Rabies virus, after a period of replication in muscles, enters the central nervous system and selectively infects certain neuronal populations; therefore, the search for other targets of RVG continues. Among these targets were neural cell adhesion molecules (NCAMs), p75 neurotrophin receptor (p75NTR), metabotropic glutamate receptor subtype 2 (mGluR 2), integrin β1 and neuronal nAChRs. It should be noted that the function of neuronal nAChR subtypes in Rabies virus infection is currently not well understood. However, the interaction was shown between full-length RVG ectodomain (as well as shorter RVG neurotoxin-like peptides) and α4β2 nAChR in vitro, which can explain the behavioral changes in *C. elegans* and mice [236]. The involvement of α7 nAChRs of the central and peripheral nervous systems in interaction with RVG is only assumed, but it has been shown that a recombinant trimeric RVG binds to α7 nAChRs expressed on monocyte-derived macrophages that induce the cholinergic anti-inflammatory pathway, characterized by a significant decrease in TNF α upon LPS challenge [237]. It has also been shown that RVG expressed in recombinant avirulent LaSota strain of Newcastle disease virus (rL-RVG) suppresses (as a competitive antagonist of α7 nAChR) the migration of gastric cancer cells by regulating α7 nAChR/ERK signaling and epithelial–mesenchymal transition [238]. A recently published cryo-EM structure of trimeric RVG complexed with RVA122, a potently neutralizing human antibody [239], should stimulate computer modeling of the RVG complexes with its targets to understand the molecular mechanism of the spread of the Rabies virus in an infected organism. 

The first studies of the interaction of nAChRs with the human immunodeficiency virus (HIV), or rather, with its envelope glycoprotein, gp120, in many ways, resembled those seen for RVG. The reason for this was the identification of a certain homology of HIV gp120 fragment 159–169 with a fragment of Rabies virus glycoprotein and the central loop of snake α-neurotoxins recognizing distinct nAChR subtypes. This fact suggested a possible interaction of gp120 with cholinoreceptors, which was confirmed for the solubilized nAChR from fetal calf muscle [240,241]. However, the main target of action for HIV gp120 soon became α7 nAChR, represented both in the nervous system and on the immune cells (see, for example, the review [242]).

The role of this receptor in relation to HIV is currently being investigated in two main lines, namely, chronic inflammation (both neuroinflammation and peripheral inflammation) and HIV-associated neurocognitive disorders. Over the past two decades, a large number of papers have been published studying the molecular mechanisms of the involvement of α7 nAChRs in these two HIV-related processes as well as looking for ways to treat or mitigate the effects of infection, most of which have been considered in recent reviews (see, for example, [243]).

With the start of the COVID-19 pandemic, it was suggested that nAChRs might also be involved, although it was quickly shown that the main target for virus entry into the host cells is angiotensin-converting enzyme 2 (ACE2), which interacts with the receptor-binding domain (RBD) of the envelope Spike protein of the virus. The “cholinergic trace” arose after observing that smokers (that is, those who consume nicotine—an exogenous non-selective agonist of various nAChR subtypes) were infected less and more easily tolerated COVID-19 infection [244]. It was later shown that nicotine causes a decrease in cytokine levels, reducing cytokine storm, which is one of the main causes of mortality in COVID-19 infection [245,246], and also acting through α7 nAChR, can demonstrate other effects [247]. However, there is still no common view about the disease process in smoking and nonsmoking patients. Thus, some studies have shown the negative effect of smoking on the development and course of COVID-19 (see, for example, a recent review [248]). In particular, it was observed that smokers with COVID-19 had a higher risk of hospitalization and death [249]. One of the explanations is that nicotine enhances the expression of ACE2, facilitating the penetration and reproduction of the SARS-CoV-2 virus [250].

It has also been shown that α-bungarotoxin blocks the increase in the amount of ACE2 induced by the action of nicotine [251], mediated through α7 nAChR. Effects similar to those of nicotine were demonstrated by GTS-21, an agonist of α7 nAChR, which, unlike nicotine, is a more selective compound, does not have toxicity and does not cause addiction and, thus, can be considered a more-promising agent for the treatment of COVID-19 [252]. It is worth noting here that one of the first drugs used in the treatment of COVID-19 was chloroquine, which also interacts effectively with the α9α10 nAChR subtype [253]. Another area of research on the relationship of COVID-19 with the cholinergic system is the possibility of direct interaction of nAChR with the S-protein of the SARS-CoV-2 virus. For the first time, such an assumption was made after the identification of a certain homology in some fragments of S-protein, with sequences of peptide antagonists of nAChRs, in particular, snake venom neurotoxins [254]. In one of the first such works, computer modeling of complexes of a number of nAChR subtypes and RBD was carried out, and the areas of their possible interaction were suggested. In particular, a high homology was revealed between the fragment (amino acid residues 375–390) of RBD of the SARS-CoV-2 virus and the snake neurotoxin NL1, which effectively interacts with nAChR [255]. In addition, according to the results of computer modeling, another region of RBD (381–386) was identified, which can form contact with the fragment (189–192) of the α9 subunit of the nAChR. Computer modeling also confirmed the high probability of the interaction of RBD with the α7 receptor subunit. Other “modelling” publications on the interaction of RBD with α7 nAChR [256] or various receptor subtypes have also appeared; for example [257], where possible, the recognition of the fragment (674–685) of the viral S-protein by three receptor subtypes-α4β2, α7 and α1β1γδ, was suggested. The experimental results concerning a possible interaction of the nAChR with the S protein fragments have appeared only very recently. In one publication [258], it was found that the immunization of mice with peptide 674–685 bound to hemocyanin resulted in decreased levels of α7 nAChRs and in higher levels of TNF-α and IL-1β. In more detail, the effects of the Y674-R685 peptide on α7 nAChRs were analyzed in [259]. Using whole-cell and single-channel recordings, its dual effects on this receptor were demonstrated: it activated the α7 nAChRs in the presence of positive allosteric modulators, thus indicating the attachment of this S-protein fragment in the vicinity of the agonist binding site but also induced a negative effect by a decrease in the duration of channel openings. However, in another recent publication [260], the authors tested the effects of the S1 domain and the entire S1–S2 ectodomain of S-protein but none of them were detected in terms of competition with radio-iodinated α-bungarotoxin for binding to the α7 nAChR heterologously expressed on the HEK-293 cells. The authors also did not see any effects of the S1 domain on the ion currents of this receptor. Apparently, further experiments are needed to elucidate a possible role, even a minor one, of nAChRs in COVID-19 disease. 

## 8. Conclusions

The reviewed recent works in the field of nAChRs have shown increasing interest in the role that α7- and α9-containing receptors play in immune processes, chronic pain and viral infections. The presented materials demonstrated the occurrence and functional role of the mentioned nAChR subtypes in different immune cells. Nicotinic ligands, from small molecules to peptides such as α-conotoxins, as well as three-finger proteins, such as snake-venom α-neurotoxins and Ly6/uPAR proteins, are excellent tools in research on various nicotinic receptors, including α7- and α9-containing nAChRs, and are considered to be potential drug leads against diseases involving inflammation and pain.

In this review, we presented extensive information on the occurrence of α7- and α9-containing nAChRs in different kinds of immune cells, illustrated the participation of these receptor subtypes in various immune functions and also discussed versatile compounds affecting these receptors. Depending on the type of immune cells and the nAChR subtype they express, different biological effects can be observed. The role of nAChRs expressed by various types of immune cells described in this review may be useful for further drug development and therapeutic strategies for the treatment of immunopathological and inflammatory diseases. This information may also be useful in selecting appropriate tools for a more thorough study of immune system functions. To follow the second topic of this Special Issue, we provided a large amount of recent data on the involvement of these nAChRs in pain relief. In accordance with this, information was presented in sufficient detail on the selective compounds, such as three-finger proteins both from snake venoms and from the Ly6/uPAR family, as well as peptide neurotoxins from marine mollusks, which can open the way to new anesthetics. We hope that this information will be useful for researchers working in this and relevant directions.

## Figures and Tables

**Table 1:** The biological effects of the actions on the α7, α9 and α9α10 nAChRs in different immune cells.

Immune Cell Type	nAChR Subtype	Agonists and Antagonists	Biological Effect	References
T-cells	α7	Nicotine	Increased FasL expression and suppressed the development of CD4^+^ T-cells	[120]
	α7	GTS-21	Enhanced the differentiation and proliferation of Tregs and effector T-cells	[122]
	α7	Nicotine	Reduced T-cell proliferation and Th1 cytokine production	[123]
	α7	Nicotine	Increased expression of CTLA-4 and Foxp3	[124]
	α9	Nicotine	Suppressed TGF-β1	[125]
	α7	GTS-21	Promoted transcription of HIV-1 proviral DNA, increased reactive oxygen species, decreased DUSP1 and DUSP6; increased p38 MAPK phosphorylation	[126]
B cells	α7	MLA	Enhanced proliferation	[131]
Dendritic cells	α7	Nicotine	Reduced production of IL-1β, IL-10, TNF-α and IL-12	[121]
	α7	SLURP-1	Attenuated cell proliferation	[134]
	α7	GTS-21	Reduced secretion of pro-inflammatory cytokines and downregulation of the CD80 and MHC II expression	[135]
	α7	GTS-21	Suppressed APC-dependent differentiation of CD4^+^ T-cells	[136]
	n.d. *	Acetylcholine	Stimulated OX40L expression, induced Th2 profile, increased production of IL-4, IL-5, and IL-13 by CD4^+^ T-cells	[137]
	n.d.	Nicotine	Increased expression of CD86 and production of less IL-12, modulation of the Th1/Th2 balance towards Th2	[138]
Monocytes	α9α10	Epibatidine	Inhibition of pro-inflammatory cytokines	[140]
	α7	Nicotine and GSK1345038	Inhibition of TNF production	[141]
	α7	Nicotine	Inhibition of IL-18-enhanced expression of ICAM-1, B7.2 and CD40 and production of IL-12, IFN-γ and TNF-α	[142]
	α7	GTS-21	Suppressed TNF-α production	[144]
	n.d.	Nicotine	Inhibition of the production of TNF-α, IL-1β and IL-12 and stimulation of the IL-10 secretion	[145]
	α9α10 and α7	Nicotine, acetylcholine, phosphocholine	Inhibition of ATP-induced release of IL-1β	[148,149]
Macrophages	n.d.	Acetylcholine	Attenuation of the release of TNF, IL-1β, IL-6 and IL-18, but not IL-10	[99]
	α7	PNU 282987	Increased expression of HLA-DR, CD11b and CD54; decreased expression of CD14 and of IL-10 production	[156]
	α7	Acetylcholine and PNU 282987	Inhibition of MMP-9 production and cell migration	[157]
		GTS-21	Inhibition of TNF-α production	[160]
Neutrophils and granulocytes	α7 and α9	Nicotine and acetylcholine	Modified respiratory burst and affected neutrophil adhesion	[168]
	α9	Nicotine and choline	Increased cell adhesion and decreased reactive oxygen species production	[169]
	n.d.	Nicotine	Release of NET, activation of Akt and PAD4	[170]
Mast cells	α7 and α9	Nicotine	Inhibition of C4 leukotriene (LTC4), TNF-α, and IL-1β	[163]
		Acetylcholine	Induced release of histamine	[164]
Natural killer cells	α7	PNU 282987	Suppressed NKG2D expression, reduced cytotoxic activity and IFN-γ production	[173]
	n.d.	Nicotine	Impairment of the ability of NK cells to kill cancer cells and release cytokines; decreased the expression of NKG2D, Ly49I and cell proliferation	[175,176]

* nAChR subtype was not determined.

## Data Availability

Not applicable.

## Abbreviations

ACE2	angiotensin-converting enzyme 2
ACh	acetylcholine
AChBP	acetylcholine-binding protein
APC	antigen-presenting cell
CAP	cholinergic anti-inflammatory pathway
CCI	chronic constriction injury
CCK	cholecystokinin
ChAT	choline acetyltransferase
CIA	collagen-induced arthritis
CIPN	chemotherapy-induced peripheral neuropathy
DC	dendritic cell
DRG	dorsal root ganglion
ECD	extracellular domain
GPI	glycosylphosphatidylinositol
HIV	human immunodeficiency virus
IFN	interferon
KO	knockout
MDM	human macrophage
MLA	methyllycaconitine
nAChR	nicotinic acetylcholine receptor
NK	natural killer
PHA	phytohemagglutinin
RVG	Rabies virus glycoprotein
RBD	receptor binding domain
SLURP-1	lymphocytic antigen-6/urokinase type plasminogen activator receptor-related peptide
Tregs	regulatory T-cells
wsLynx1	water-soluble Lynx1
